# Supplementary material for: The mHealth clinical decision-making tools for maternal and perinatal health care in Sub-Saharan Africa: A systematic review
Source: PLoS One. 2025 Apr 24;20(4):e0319510. doi: 10.1371/journal.pone.0319510 (PMC12021198; doi:10.1371/journal.pone.0319510)
Supplement: S1 File — This is the S1 file Appendix 1: Search strategies. (PDF) [file pone.0319510.s001.pdf]

Appendix 1: Search strategies

The Boolean term ‘OR’ is used between columns, with ‘AND’ being used between rows. This gives each search four terms: broadly, (mHealth) AND (maternal and perinatal care) AND (decision-making) AND (Sub-Saharan Africa).

**PubMed**	
**Mesh terms**	**Other terms**
Cell phone; cell phone use; telemedicine; mobile applications; computers, handheld "Cell Phone"[Mesh] OR "Cell Phone Use"[Mesh] OR "Telemedicine"[Mesh] OR "Mobile Applications"[Mesh] OR "Computers, Handheld"[Mesh]	mobile phone* or mobile* or m-health* or mobile health* or mhealth* or m health* or electronic* or smartphone* or smart phone* or iPhone* or iPad* or tablet computer* or cellphone* or electronic health* or eHealth* or e-health* or cell phone* or handheld computer* or phone* or mobile device*
Pregnancy; prenatal care; perinatal care; postnatal care; parturition; maternal health; maternal health services; “Pregnancy"[Mesh] OR "Prenatal Care"[Mesh] OR "Perinatal Care"[Mesh] OR "Postnatal Care"[Mesh] OR "Maternal Health Services"[Mesh] OR "Maternal Health"[Mesh]	Parturition* or childbirth* or labour* or maternal* or prenatal* or perinatal* or antenatal* or postnatal* or pregnancy* or maternal health* or obstetrics* or intrapartum* or antepartum* or postpartum*
clinical decision-making; decision making; decision support systems, clinical; “Clinical Decision-Making"[Mesh] OR "Decision Support Systems, Clinical"[Mesh] OR "Decision Making"[Mesh]	Decision-making* or decision making* or decision-support* or decision support*
"Africa South of the Sahara"[Mesh]	Sub Saharan Africa* or sub-Saharan Africa* or Angola* or Benin* or Botswana* or Burkina Faso* or Burundi* or Cabo Verde* or Cameroon* or Central African Republic* or Chad* or Comoros* or Congo* or Cote

	D Ivoire* or Democratic Republic of the Congo* or Equatorial Guinea* or Eritrea* or Eswatini* or Ethiopia* or Gabon* or Gambia* or Ghana* or Guinea* or Guinea-Bissau* or Kenya* or Lesotho* or Liberia* or Madagascar* or Malawi* or Mali* or Mauritania* or Mauritius* or Mozambique* or Namibia* or Niger* or Nigeria* or Rwanda* or “Sao Tome and Principe”* or Senegal* or Seychelles* or Sierra Leone* or Somalia* or South Africa* or South Sudan* or Sudan* or Tanzania* or Togo* or Uganda* or Zambia* or Zimbabwe*

((("mobile"[All Fields] OR "mobiles"[All Fields]) AND "phone*"[All Fields]) OR "mobile*"[All Fields] OR "m health*"[All Fields] OR (("mobile"[All Fields] OR "mobiles"[All Fields]) AND "health*"[All Fields]) OR "mhealth*"[All Fields] OR ("m"[All Fields] AND "health*"[All Fields]) OR "electronic*"[All Fields] OR "smartphone*"[All Fields] OR (("smart"[All Fields] OR "smartness"[All Fields] OR "smarts"[All Fields]) AND "phone*"[All Fields]) OR "iphone*"[All Fields] OR "ipad*"[All Fields] OR (("tablet s"[All Fields] OR "tabletability"[All Fields] OR "tableted"[All Fields] OR "tablets"[MeSH Terms] OR "tablets"[All Fields] OR "tablet"[All Fields] OR "tableting"[All Fields] OR "tabletted"[All Fields] OR "tabletting"[All Fields]) AND "computer*"[All Fields]) OR "cellphone*"[All Fields] OR (("electronical"[All Fields] OR "electronically"[All Fields] OR "electronics"[MeSH Terms] OR "electronics"[All Fields] OR "electronic"[All Fields]) AND "health*"[All Fields]) OR "ehealth*"[All Fields] OR "e health*"[All Fields] OR (("cells"[MeSH Terms] OR "cells"[All Fields] OR "cell"[All Fields]) AND "phone*"[All Fields]) OR (("handheld"[All Fields] OR "handhelds"[All Fields]) AND "computer*"[All Fields]) OR "phone*"[All Fields] OR (("mobile"[All Fields] OR "mobiles"[All Fields]) AND "device*"[All Fields]) OR ("Cell Phone"[MeSH Terms] OR "Cell Phone Use"[MeSH Terms] OR "Telemedicine"[MeSH Terms] OR "Mobile Applications"[MeSH Terms] OR "computers, handheld"[MeSH Terms])) AND ("Pregnancy"[MeSH Terms] OR "Prenatal Care"[MeSH Terms] OR "Perinatal Care"[MeSH Terms] OR "Postnatal Care"[MeSH Terms] OR

"Maternal Health Services"[MeSH Terms] OR "Maternal Health"[MeSH Terms] OR ("parturition*"[All Fields] OR "childbirth*"[All Fields] OR "labour*"[All Fields] OR "maternal*"[All Fields] OR "prenatal*"[All Fields] OR "perinatal*"[All Fields] OR "antenatal*"[All Fields] OR "postnatal*"[All Fields] OR "pregnancy*"[All Fields] OR (("maternally"[All Fields] OR "maternities"[All Fields] OR "maternity"[All Fields] OR "mothers"[MeSH Terms] OR "mothers"[All Fields] OR "maternal"[All Fields]) AND "health*"[All Fields]) OR "obstetrics*"[All Fields] OR "intrapartum*"[All Fields] OR "antepartum*"[All Fields] OR "postpartum*"[All Fields])) AND ("Clinical Decision-Making"[MeSH Terms] OR "decision support systems, clinical"[MeSH Terms] OR "Decision Making"[MeSH Terms] OR ("decision making*"[All Fields] OR (("decision"[All Fields] OR "decision s"[All Fields] OR "decisions"[All Fields] OR "decisive"[All Fields] OR "decisively"[All Fields]) AND "making*"[All Fields]) OR "decision support*"[All Fields] OR (("decision"[All Fields] OR "decision s"[All Fields] OR "decisions"[All Fields] OR "decisive"[All Fields] OR "decisively"[All Fields]) AND "support*"[All Fields]))) AND ("Africa South of the Sahara"[MeSH Terms] OR (("Sub"[All Fields] AND ("saharan"[All Fields] OR "saharans"[All Fields]) AND "africa*"[All Fields]) OR ("sub-Saharan"[All Fields] AND "africa*"[All Fields]) OR "angola*"[All Fields] OR "benin*"[All Fields] OR "botswana*"[All Fields] OR ("Burkina"[All Fields] AND "faso*"[All Fields]) OR "burundi*"[All Fields] OR (("cabo"[Journal] OR "cabo"[All Fields]) AND "verde*"[All Fields]) OR "cameroon*"[All Fields] OR (("central"[All Fields] OR "centrally"[All Fields] OR "centrals"[All Fields]) AND ("african people"[MeSH Terms] OR ("african"[All Fields] AND "people"[All Fields]) OR "african people"[All Fields] OR "africans"[All Fields] OR "black people"[MeSH Terms] OR ("black"[All Fields] AND "people"[All Fields]) OR "black people"[All Fields] OR "african"[All Fields]) AND "republic*"[All Fields]) OR "chad*"[All Fields] OR "comoros*"[All Fields] OR "congo*"[All Fields] OR ((cote d[Author] OR cote d[Investigator]) AND "ivoire*"[All Fields]) OR (("democrat"[All Fields] OR "democratic"[All Fields] OR "democratically"[All Fields] OR "democratization"[All Fields] OR "democratize"[All Fields] OR "democratized"[All Fields] OR "democratizing"[All Fields] OR "democrats"[All Fields]) AND ("republic"[All Fields] OR "republic s"[All Fields] OR "republics"[All Fields]) AND "congo*"[All Fields]) OR (("equatorial"[All Fields] OR "equatorially"[All Fields] OR "equatorials"[All Fields]) AND "guinea*"[All Fields]) OR "eritrea*"[All Fields] OR "eswatini*"[All Fields] OR "ethiopia*"[All Fields] OR "gabon*"[All Fields] OR "gambia*"[All Fields] OR "ghana*"[All Fields] OR "guinea*"[All Fields] OR "guinea bissau*"[All Fields] OR "kenya*"[All Fields] OR "lesotho*"[All Fields]

OR "liberia*"[All Fields] OR "madagascar*"[All Fields] OR "malawi*"[All Fields] OR "mali*"[All Fields] OR "mauritania*"[All Fields] OR "mauritius*"[All Fields] OR "mozambique*"[All Fields] OR "namibia*"[All Fields] OR "niger*"[All Fields] OR "nigeria*"[All Fields] OR "rwanda*"[All Fields] OR "Sao Tome and Principe"[All Fields] OR "senegal*"[All Fields] OR "seychelles*"[All Fields] OR (("sierra"[All Fields] OR "sierras"[All Fields]) AND "leone*"[All Fields]) OR "somalia*"[All Fields] OR ("South"[All Fields] AND "africa*"[All Fields]) OR ("South"[All Fields] AND "sudan*"[All Fields]) OR "sudan*"[All Fields] OR "tanzania*"[All Fields] OR "togo*"[All Fields] OR "uganda*"[All Fields] OR "zambia*"[All Fields] OR "zimbabwe*"[All Fields]))

**CINAHL**	
**Cinahl Subject Headings (MH)**	**Other terms**
Mobile applications; computers, handheld; cellular phone; telehealth	mobile phone* or mobile* or m-health* or mobile health* or mhealth* or m health* or electronic* or smartphone* or smart phone* or iPhone* or iPad* or tablet computer* or cellphone* or electronic health* or ehealth* or e-health* or cell phone* or handheld computer* or phone* or mobile device*
Maternal health services; prenatal care; perinatal care; postnatal care; pregnancy;	Parturition* or childbirth* or labour* or maternal* or prenatal* or perinatal* or antenatal* or postnatal* or pregnancy* or maternal health* or obstetrics* or intrapartum* or antepartum* or postpartum*
Decision making; decision making, clinical; decision support systems, clinical;	Decision-making* or decision making* or decision-support* or decision support*
Africa South of the Sahara	Sub Saharan Africa* or sub-Saharan Africa* or Angola* or Benin* or Botswana* or Burkina Faso* or Burundi* or Cabo Verde* or Cameroon* or Central African Republic* or Chad* or Comoros* or Congo* or Cote D Ivoire* or Democratic Republic of the Congo* or Equatorial

	Guinea* or Eritrea* or Eswatini* or Ethiopia* or Gabon* or Gambia* or Ghana* or Guinea* or Guinea-Bissau* or Kenya* or Lesotho* or Liberia* or Madagascar* or Malawi* or Mali* or Mauritania* or Mauritius* or Mozambique* or Namibia* or Niger* or Nigeria* or Rwanda* or "Sao Tome and Principe"* or Senegal* or Seychelles* or Sierra Leone* or Somalia* or South Africa* or South Sudan* or Sudan* or Tanzania* or Togo* or Uganda* or Zambia* or Zimbabwe*
**GLOBAL HEALTH**	
**Global Health Subject Headings**	**Other terms**
Mobile telephones; mobile applications; mHealth;	mobile phone* or mobile* or m-health* or mobile health* or mhealth* or m health* or electronic* or smartphone* or smart phone* or iPhone* or iPad* or tablet computer* or cellphone* or electronic health* or ehealth* or e-health* or cell phone* or handheld computer* or phone* or mobile device*
Prenatal care; pregnancy; puerperium; childbirth;	Parturition* or childbirth* or labour* or maternal* or prenatal* or perinatal* or antenatal* or postnatal* or pregnancy* or maternal health* or obstetrics* or intrapartum* or antepartum* or postpartum*
Decision making;	Decision-making* or decision making* or decision-support* or decision support*
Africa South of Sahara;	Sub-Saharan Africa* or Angola* or Benin* or Botswana* or Burkina Faso* or Burundi* or Cabo Verde* or Cameroon* or Central African Republic* or Chad* or Comoros* or Congo* or Cote D Ivoire* or Democratic Republic of the Congo* or Equatorial Guinea* or Eritrea* or Eswatini* or Ethiopia* or Gabon* or Gambia* or Ghana* or Guinea* or Guinea-Bissau* or Kenya* or Lesotho* or Liberia* or Madagascar* or Malawi* or Mali* or

	Mauritania* or Mauritius* or Mozambique* or Namibia* or Niger* or Nigeria* or Rwanda* or (Sao Tome and Principe) or Senegal* or Seychelles* or Sierra Leone* or Somalia* or South Africa* or South Sudan* or Sudan* or Tanzania* or Togo* or Uganda* or Zambia* or Zimbabwe*
**EMBASE**	
**EMBASE Subject Headings**	**Other terms**
Mobile application; mobile health application; mobile phone;	mobile phone* or mobile* or m-health* or mobile health* or mhealth* or m health* or electronic* or smartphone* or smart phone* or iPhone* or iPad* or tablet computer* or cellphone* or electronic health* or ehealth* or e-health* or cell phone* or handheld computer* or phone* or mobile device*
Prenatal care; intrapartum care; postnatal care; childbirth; pregnancy; maternal health service;	Parturition* or childbirth* or labour* or maternal* or prenatal* or perinatal* or antenatal* or postnatal* or pregnancy* or maternal health* or obstetrics* or intrapartum* or antepartum* or postpartum*
Decision making; clinical decision making; clinical decision support system; decision support system; medical decision making	Decision-making* or decision making* or decision-support* or decision support*
Africa south of the Sahara;	Sub-Saharan Africa* or Angola* or Benin* or Botswana* or Burkina Faso* or Burundi* or Cabo Verde* or Cameroon* or Central African Republic* or Chad* or Comoros* or Congo* or Cote D Ivoire* or Democratic Republic of the Congo* or Equatorial Guinea* or Eritrea* or Eswatini* or Ethiopia* or Gabon* or Gambia* or Ghana* or Guinea* or Guinea-Bissau* or Kenya* or Lesotho* or Liberia* or Madagascar* or Malawi* or Mali* or Mauritania* or Mauritius* or Mozambique* or Namibia* or Niger* or Nigeria* or Rwanda* or (Sao

	Tome and Principe) or Senegal* or Seychelles* or Sierra Leone* or Somalia* or South Africa* or South Sudan* or Sudan* or Tanzania* or Togo* or Uganda* or Zambia* or Zimbabwe*
**Web of science**	
**Terms used**	
mobile phone* or mobile* or m-health* or mobile health* or mhealth* or m health* or electronic* or smartphone* or smart phone* or iPhone* or iPad* or tablet computer* or cellphone* or electronic health* or ehealth* or e-health* or cell phone* or handheld computer* or phone* or mobile device*	
Parturition* or childbirth* or labour* or maternal* or prenatal* or perinatal* or antenatal* or postnatal* or pregnancy* or maternal health* or obstetrics* or intrapartum* or antepartum* or postpartum*	
Decision-making* or decision making* or decision-support* or decision support*	
Sub-Saharan Africa* or Angola* or Benin* or Botswana* or Burkina Faso* or Burundi* or Cabo Verde* or Cameroon* or Central African Republic* or Chad* or Comoros* or Congo* or Cote D Ivoire* or Democratic Republic of the Congo* or Equatorial Guinea* or Eritrea* or Eswatini* or Ethiopia* or Gabon* or Gambia* or Ghana* or Guinea* or Guinea-Bissau* or Kenya* or Lesotho* or Liberia* or Madagascar* or Malawi* or Mali* or Mauritania* or Mauritius* or Mozambique* or Namibia* or Niger* or Nigeria* or Rwanda* or (Sao Tome and Principe) or Senegal* or Seychelles* or Sierra Leone* or Somalia* or South Africa* or South Sudan* or Sudan* or Tanzania* or Togo* or Uganda* or Zambia* or Zimbabwe*	

Grey literature sources searched:

The sources for the grey literature search were compiled from searching the methodology of other systematic reviews on mHealth use in low resource settings.

African Strategies for Health www.africanstrategies4health.org

BBC Media Action https://www.bbc.co.uk/mediaaction

D-Tree International https://www.d-tree.org

GMSA https://www.gsma.com

Jhpiego https://www.jhpiego.org

Maternal Health Task Force https://www.mhtf.org

Maternity Worldwide https://www.maternityworldwide.org

Mothers2mothers https://m2m.org

Partners in Health https://www.pih.org/#

PharmAccess https://www.pharmaccess.org

Proquest Dissertations and Theses Global http://proquest.com/pqdtglobal

Thrive https://www.thriveagency.uk

USAID https://www.usaid.gov

WHO https://apps.who.int/iris/

World Bank https://www.worldbank.org/en/home
