# Supplementary material for: The mHealth clinical decision-making tools for maternal and perinatal health care in Sub-Saharan Africa: A systematic review
Source: PLoS One. 2025 Apr 24;20(4):e0319510. doi: 10.1371/journal.pone.0319510 (PMC12021198; doi:10.1371/journal.pone.0319510)
Supplement: S2 File — (PDF) [file pone.0319510.s002.pdf]

Appendix 2

Revised Cochrane risk-of-bias tool for randomized trials (RoB 2) TEMPLATE FOR COMPLETION

Edited by Julian PT Higgins, Jelena Savović, Matthew J Page, Jonathan AC Sterne
on behalf of the RoB2 Development Group

Version of 22 August 2019

The development of the RoB 2 tool was supported by the MRC Network of Hubs for Trials Methodology Research (MR/L004933/2- N61), with the support of the host MRC ConDuCT-II Hub (Collaboration and innovation for Difficult and Complex randomised controlled Trials In Invasive procedures - MR/K025643/1), by MRC research grant MR/M025209/1, and by a grant from The Cochrane Collaboration.

This work is licensed under a Creative Commons Attribution-NonCommercial-NoDerivatives 4.0 International License.

Study details

Reference

Study design

- X Individually-randomized parallel-group trial
- ☐ Cluster-randomized parallel-group trial
- ☐ Individually randomized cross-over (or other matched) trial

For the purposes of this assessment, the interventions being compared are defined as

Experimental: ______ Comparator: ______

**Specify which outcome is being assessed for risk of bias**	
**Specify the numerical result being assessed.** In case of multiple alternative analyses being presented, specify the numeric result (e.g. RR = 1.52 (95% CI 0.83 to 2.77) and/or a reference (e.g. to a table, figure or paragraph) that uniquely defines the result being assessed.	

Is the review team's aim for this result…?

☐ to assess the effect of *assignment to intervention* (the 'intention-to-treat' effect)

☐ to assess the effect of *adhering to intervention* (the 'per-protocol' effect)

If the aim is to assess the effect of *adhering to intervention*, select the deviations from intended intervention that should be addressed (at least one must be checked):

☐ occurrence of non-protocol interventions

☐ failures in implementing the intervention that could have affected the outcome

☐ non-adherence to their assigned intervention by trial participants

Which of the following sources were <u>obtained</u> to help inform the risk-of-bias assessment? (tick as many as apply)

☐ Journal article(s) with results of the trial

☐ Trial protocol

☐ Statistical analysis plan (SAP)

☐ Non-commercial trial registry record (e.g. ClinicalTrials.gov record)

☐ Company-owned trial registry record (e.g. GSK Clinical Study Register record)

☐ "Grey literature" (e.g. unpublished thesis)

☐ Conference abstract(s) about the trial

☐ Regulatory document (e.g. Clinical Study Report, Drug Approval Package)

☐ Research ethics application

☐ Grant database summary (e.g. NIH RePORTER or Research Councils UK Gateway to Research)

☐ Personal communication with trialist

☐ Personal communication with the sponsor

Risk of bias assessment

Responses underlined in green are potential markers for low risk of bias, and responses in red are potential markers for a risk of bias. Where questions relate only to sign posts to other questions, no formatting is used.

Domain 1: Risk of bias arising from the randomization process

Signalling questions	Comments	Response options
**1.1 Was the allocation sequence random?**		Y / PY / PN / N / NI
**1.2 Was the allocation sequence concealed until participants were enrolled and assigned to interventions?**		Y / PY / PN / N / NI
**1.3 Did baseline differences between intervention groups suggest a problem with the randomization process?**		Y / PY / PN / N / NI
**Risk-of-bias judgement**		Low / High / Some concerns
Optional: What is the predicted direction of bias arising from the randomization process?		NA / Favours experimental / Favours comparator / Towards null /Away from null / Unpredictable

Domain 2: Risk of bias due to deviations from the intended interventions (*effect of assignment to intervention*)

Signalling questions	Comments	Response options
**2.1. Were participants aware of their assigned intervention during the trial?**		Y / PY / PN / N / NI
**2.2. Were carers and people delivering the interventions aware of participants' assigned intervention during the trial?**		Y / PY / PN / N / NI
**2.3. If Y/PY/NI to 2.1 or 2.2: Were there deviations from the intended intervention that arose because of the trial context?**		NA / Y / PY / PN / N / NI
**2.4 If Y/PY to 2.3: Were these deviations likely to have affected the outcome?**		NA / Y / PY / PN / N / NI
**2.5. If Y/PY/NI to 2.4: Were these deviations from intended intervention balanced between groups?**		NA / Y / PY / PN / N / NI
**2.6 Was an appropriate analysis used to estimate the effect of assignment to intervention?**		Y / PY / PN / N / NI
**2.7 If N/PN/NI to 2.6: Was there potential for a substantial impact (on the result) of the failure to analyse participants in the group to which they were randomized?**		NA / Y / PY / PN / N / NI
**Risk-of-bias judgement**		Low / High / Some concerns
Optional: What is the predicted direction of bias due to deviations from intended interventions?		NA / Favours experimental / Favours comparator / Towards null /Away from null / Unpredictable

Domain 2: Risk of bias due to deviations from the intended interventions (*effect of adhering to intervention*)

Signalling questions	Comments	Response options
**2.1. Were participants aware of their assigned intervention during the trial?**		Y / PY / PN / N / NI
**2.2. Were carers and people delivering the interventions aware of participants' assigned intervention during the trial?**		Y / PY / PN / N / NI
**2.3. [If applicable:] If Y/PY/NI to 2.1 or 2.2: Were important non-protocol interventions balanced across intervention groups?**		NA / Y / PY / PN / N / NI
**2.4. [If applicable:] Were there failures in implementing the intervention that could have affected the outcome?**		NA / Y / PY / PN / N / NI
**2.5. [If applicable:] Was there non-adherence to the assigned intervention regimen that could have affected participants' outcomes?**		NA / Y / PY / PN / N / NI
**2.6. If N/PN/NI to 2.3, or Y/PY/NI to 2.4 or 2.5: Was an appropriate analysis used to estimate the effect of adhering to the intervention?**		NA / Y / PY / PN / N / NI
**Risk-of-bias judgement**		Low / High / Some concerns
Optional: What is the predicted direction of bias due to deviations from intended interventions?		NA / Favours experimental / Favours comparator / Towards null /Away from null / Unpredictable

Domain 3: Missing outcome data

Signalling questions	Comments	Response options
**3.1 Were data for this outcome available for all, or nearly all, participants randomized?**		Y / PY / PN / N / NI
**3.2 If N/PN/NI to 3.1: Is there evidence that the result was not biased by missing outcome data?**		NA / Y / PY / PN / N
**3.3 If N/PN to 3.2: Could missingness in the outcome depend on its true value?**		NA / Y / PY / PN / N / NI
**3.4 If Y/PY/NI to 3.3: Is it likely that missingness in the outcome depended on its true value?**		NA / Y / PY / PN / N / NI
**Risk-of-bias judgement**		Low / High / Some concerns
Optional: What is the predicted direction of bias due to missing outcome data?		NA / Favours experimental / Favours comparator / Towards null /Away from null / Unpredictable

Domain 4: Risk of bias in measurement of the outcome

Signalling questions	Comments	Response options
**4.1 Was the method of measuring the outcome inappropriate?**		Y / PY / PN / N / NI
**4.2 Could measurement or ascertainment of the outcome have differed between intervention groups?**		Y / PY / PN / N / NI
**4.3 If N/PN/NI to 4.1 and 4.2: Were outcome assessors aware of the intervention received by study participants?**		NA / Y / PY / PN / N / NI
**4.4 If Y/PY/NI to 4.3: Could assessment of the outcome have been influenced by knowledge of intervention received?**		NA / Y / PY / PN / N / NI
**4.5 If Y/PY/NI to 4.4: Is it likely that assessment of the outcome was influenced by knowledge of intervention received?**		NA / Y / PY / PN / N / NI
**Risk-of-bias judgement**		Low / High / Some concerns
Optional: What is the predicted direction of bias in measurement of the outcome?		NA / Favours experimental / Favours comparator / Towards null /Away from null / Unpredictable

Domain 5: Risk of bias in selection of the reported result

Signalling questions	Comments	Response options
**5.1 Were the data that produced this result analysed in accordance with a pre-specified analysis plan that was finalized before unblinded outcome data were available for analysis?**		Y / PY / PN / N / NI
**Is the numerical result being assessed likely to have been selected, on the basis of the results, from...**		
**5.2. ... multiple eligible outcome measurements (e.g. scales, definitions, time points) within the outcome domain?**		Y / PY / PN / N / NI
**5.3 ... multiple eligible analyses of the data?**		Y / PY / PN / N / NI
**Risk-of-bias judgement**		Low / High / Some concerns
Optional: What is the predicted direction of bias due to selection of the reported result?		NA / Favours experimental / Favours comparator / Towards null /Away from null / Unpredictable

Overall risk of bias

**Risk-of-bias judgement**		Low / High / Some concerns
Optional: What is the overall predicted direction of bias for this outcome?		NA / Favours experimental / Favours comparator / Towards null /Away from null / Unpredictable

This work is licensed under a Creative Commons Attribution-Non-Commercial-No Derivatives 4.0 International License

Revised Cochrane risk-of-bias tool for cluster-randomized trials (RoB 2 CRT)
TEMPLATE FOR COMPLETION

Version of 18 March 2021

The development of the RoB 2 tool was supported by the MRC Network of Hubs for Trials Methodology Research (MR/L004933/2- N61), with the support of the host MRC ConDuCT-II Hub (Collaboration and innovation for Difficult and Complex randomised controlled Trials In Invasive procedures - MR/K025643/1), by MRC research grant MR/M025209/1, and by a grant from The Cochrane Collaboration.

This work is licensed under a Creative Commons Attribution-NonCommercial-NoDerivatives 4.0 International License.

Study details

**Reference**	

Study design

☐ Individually-randomized parallel-group trial
X Cluster-randomized parallel-group trial
☐ Individually randomized cross-over (or other matched) trial

For the purposes of this assessment, the interventions being compared are defined as

Experimental: [ ] Comparator: [ ]

Specify which outcome is being assessed for risk of bias [ ]

Specify the numerical result being assessed. In case of multiple alternative analyses being presented, specify the numeric result (e.g. RR = 1.52 (95% CI 0.83 to 2.77) and/or a reference (e.g. to a table, figure or paragraph) that uniquely defines the result being assessed. [ ]

Is the review team's aim for this result…?

☐ to assess the effect of *assignment to intervention* (the 'intention-to-treat' effect)
☐ to assess the effect of *adhering to intervention* (the 'per-protocol' effect)

If the aim is to assess the effect of *adhering to intervention*, select the deviations from intended intervention that should be addressed (at least one must be checked):

☐ occurrence of non-protocol interventions
☐ failures in implementing the intervention that could have affected the outcome
☐ non-adherence to their assigned intervention by trial participants

Which of the following sources were <u>obtained</u> to help inform the risk-of-bias assessment? (tick as many as apply)

☐ Journal article(s) with results of the trial
☐ Trial protocol
☐ Statistical analysis plan (SAP)
☐ Non-commercial trial registry record (e.g. ClinicalTrials.gov record)
☐ Company-owned trial registry record (e.g. GSK Clinical Study Register record)
☐ "Grey literature" (e.g. unpublished thesis)
☐ Conference abstract(s) about the trial
☐ Regulatory document (e.g. Clinical Study Report, Drug Approval Package)
☐ Research ethics application
☐ Grant database summary (e.g. NIH RePORTER or Research Councils UK Gateway to Research)
☐ Personal communication with trialist

☐	Personal communication with the sponsor

Risk of bias assessment

Responses underlined in green are potential markers for low risk of bias, and responses in red are potential markers for a risk of bias. Where questions relate only to sign posts to other questions, no formatting is used.

Domain 1a: Risk of bias arising from the randomization process

**Signalling questions**	**Comments**	**Response options**
**1a.1 Was the allocation sequence random?**		Y / PY / PN / N / NI
**1a.2 Was the allocation sequence concealed until clusters were enrolled and assigned to interventions?**		Y / PY / PN / N / NI
**1a.3 Did baseline differences between intervention groups suggest a problem with the randomization process?**		Y / PY / PN / N / NI
**Risk-of-bias judgement**		Low / High / Some concerns
Optional: What is the predicted direction of bias arising from the randomization process?		NA / Favours experimental / Favours comparator / Towards null /Away from null / Unpredictable

Domain 1b: Risk of bias arising from the timing of identification or recruitment of participants in a cluster-randomized trial

Signalling questions	Comments	Response options
**1b.1 Were all the individual participants identified and recruited (if appropriate) before randomization of clusters?**		Y/PY/PN/N/NI
**1b.2 If N/PN/NI to 1b.1: Is it likely that selection of individual participants was affected by knowledge of the intervention assigned to the cluster?**		NA/Y/PY/PN/N/NI
**1b.3 Were there baseline imbalances that suggest differential identification or recruitment of individual participants between intervention groups?**		Y/PY/PN/N/NI
**Risk-of-bias judgement**		Low / High / Some concerns
Optional: What is the predicted direction of bias arising from the timing of identification and recruitment of participants?		NA / Favours experimental / Favours comparator / Towards null /Away from null / Unpredictable

Domain 2: Risk of bias due to deviations from the intended interventions (*effect of assignment to intervention*)

Signalling questions	Comments	Response options
**2.1a Were participants aware that they were in a trial?**		Y / PY / PN / N / NI
**2.1b. If Y/PY/NI to 2.1a: Were participants aware of their assigned intervention during the trial?**		NA / Y / PY / PN / N / NI
**2.2. Were carers and people delivering the interventions aware of participants' assigned intervention during the trial?**		Y / PY / PN / N / NI
**2.3. If Y/PY/NI to 2.1 or 2.2: Were there deviations from the intended intervention that arose because of the trial context?**		NA / Y / PY / PN / N / NI
**2.4 If Y/PY to 2.3: Were these deviations likely to have affected the outcome?**		NA / Y / PY / PN / N / NI
**2.5. If Y/PY/NI to 2.4: Were these deviations from intended intervention balanced between groups?**		NA / Y / PY / PN / N / NI
**2.6 Was an appropriate analysis used to estimate the effect of assignment to intervention?**		Y / PY / PN / N / NI
**2.7 If N/PN/NI to 2.6: Was there potential for a substantial impact (on the result) of the failure to analyse participants in the group to which they were randomized?**		NA / Y / PY / PN / N / NI
**Risk-of-bias judgement**		Low / High / Some concerns
Optional: What is the predicted direction of bias due to deviations from intended interventions?		NA / Favours experimental / Favours comparator / Towards null /Away from null / Unpredictable

Domain 2: Risk of bias due to deviations from the intended interventions (*effect of adhering to intervention*)

Signalling questions	Comments	Response options
**2.1. Were participants aware of their assigned intervention during the trial?**		Y / PY / PN / N / NI
**2.2. Were carers and people delivering the interventions aware of participants' assigned intervention during the trial?**		Y / PY / PN / N / NI
**2.3. [If applicable:] If Y/PY/NI to 2.1 or 2.2: Were important non-protocol interventions balanced across intervention groups?**		NA / Y / PY / PN / N / NI
**2.4. [If applicable:] Were there failures in implementing the intervention that could have affected the outcome?**		NA / Y / PY / PN / N / NI
**2.5. [If applicable:] Was there non-adherence to the assigned intervention regimen that could have affected participants' outcomes?**		NA / Y / PY / PN / N / NI
**2.6. If N/PN/NI to 2.3, or Y/PY/NI to 2.4 or 2.5: Was an appropriate analysis used to estimate the effect of adhering to the intervention?**		NA / Y / PY / PN / N / NI
**Risk-of-bias judgement**		Low / High / Some concerns
Optional: What is the predicted direction of bias due to deviations from intended interventions?		NA / Favours experimental / Favours comparator / Towards null /Away from null / Unpredictable

Domain 3: Risk of bias due to missing outcome data

Signalling questions	Comments	Response options
**3.1a Were data for this outcome available for all clusters that recruited participants?**		Y / PY / PN / N / NI
**3.1b Were data for this outcome available for all, or nearly all, participants within clusters?**		Y / PY / PN / N / NI
**3.2 If N/PN/NI to 3.1a or 3.1b: Is there evidence that the result was not biased by missing data?**		NA / Y / PY / PN / N
**3.3 If N/PN to 3.2 Could missingness in the outcome depend on its true value?**		NA / Y / PY / PN / N / NI
**3.4 If Y/PY/NI to 3.3: Is it likely that missingness in the outcome depended on its true value?**		NA / Y / PY / PN / N / NI
**Risk-of-bias judgement**		Low / High / Some concerns
Optional: What is the predicted direction of bias due to missing outcome data?		NA / Favours experimental / Favours comparator / Towards null /Away from null / Unpredictable

Domain 4: Risk of bias in measurement of the outcome

Signalling questions	Comments	Response options
**4.1 Was the method of measuring the outcome inappropriate?**		Y / PY / PN / N / NI
**4.2 Could measurement or ascertainment of the outcome have differed between intervention groups?**		Y / PY / PN / N / NI
**4.3a If N/PN/NI to 4.1 and 4.2: Were outcome assessors aware that a trial was taking place?**		NA / Y / PY / PN / N / NI
**4.3b If Y/PY/NI to 4.3a: Were outcome assessors aware of the intervention received by study participants?**		NA / Y / PY / PN / N / NI
**4.4 If Y/PY/NI to 4.3b: Could assessment of the outcome have been influenced by knowledge of intervention received?**		NA / Y / PY / PN / N / NI
**4.5 If Y/PY/NI to 4.4: Is it likely that assessment of the outcome was influenced by knowledge of intervention received?**		NA / Y / PY / PN / N / NI
**Risk-of-bias judgement**		Low / High / Some concerns
Optional: What is the predicted direction of bias in measurement of the outcome?		NA / Favours experimental / Favours comparator / Towards null /Away from null / Unpredictable

Domain 5: Risk of bias in selection of the reported result

Signalling questions	Comments	Response options
**5.1 Were the data that produced this result analysed in accordance with a pre-specified analysis plan that was finalized before unblinded outcome data were available for analysis?**		Y / PY / PN / N / NI
**Is the numerical result being assessed likely to have been selected, on the basis of the results, from...**		
**5.2. ... multiple eligible outcome measurements (e.g. scales, definitions, time points) within the outcome domain?**		Y / PY / PN / N / NI
**5.3 ... multiple eligible analyses of the data?**		Y / PY / PN / N / NI
**Risk-of-bias judgement**		Low / High / Some concerns
Optional: What is the predicted direction of bias due to selection of the reported result?		NA / Favours experimental / Favours comparator / Towards null /Away from null / Unpredictable

Overall risk of bias

**Risk-of-bias judgement**		Low / High / Some concerns
Optional: What is the overall predicted direction of bias for this outcome?		NA / Favours experimental / Favours comparator / Towards null /Away from null / Unpredictable

This work is licensed under a Creative Commons Attribution-NonCommercial-NoDerivatives 4.0 International License
