# Supplementary material for: The mHealth clinical decision-making tools for maternal and perinatal health care in Sub-Saharan Africa: A systematic review
Source: PLoS One. 2025 Apr 24;20(4):e0319510. doi: 10.1371/journal.pone.0319510 (PMC12021198; doi:10.1371/journal.pone.0319510)
Supplement: S3 File — (PDF) [file pone.0319510.s003.pdf]

**Table 3. Risk of bias and quality/ assessment table**

| SN | Study design | Title, Author, Year | Assessment tool used | Overall scores | Risk of bias level (Low/Moderate/ High | Comment(s) (specify reasons for risk) |
|---|---|---|---|---|---|---|
| 1 | Cluster randomised controlled trial | The ability and safety of community-based health workers to safely initiate lifesaving therapies for pre-eclampsia in Ogun State, Nigeria: An analysis of 260 community treatments with MgSO4 and/or methyldopa/Adepoju et al, 2021 | Cochrane Collaboration's Risk of Bias Tool for Cluster Randomized Trials | No numeric scoring | Moderate risk | Some issues like missing follow-up data and potential baseline imbalance |
| 2 | Cluster randomised controlled trial | Community-level interventions for pre-eclampsia (CLIP) in Mozambique: A cluster randomised controlled trial/Sevene et al, 2020 | Cochrane Collaboration's Risk of Bias Tool for Cluster Randomized Trials | No numeric scoring | Moderate risk | Lack of blinding and baseline imbalance |
| 3 | Cluster randomised controlled trial | Impact of smartphone-assisted prenatal home visits on women's use of facility delivery: Results from a cluster-randomised trial in rural Tanzania/Hackett et al, 2018 | Cochrane Collaboration's Risk of Bias Tool for Cluster Randomized Trials | No numeric scoring | Moderate risk | Lack of blinding and baseline imbalance |
| 4 | Cross-sectional design | Improving the Quality of Antenatal Care Using Mobile Health in Madagascar: Five-Year Cross-Sectional Study/Benski et al, 2020 | Modified NOS | 8/10 | Low risk | It's a cross-sectional design, we used Modified NOS |
| 5 | Mixed methods evaluation | mHealth for Safer Deliveries: A mixed methods evaluation of the effect of an integrated mobile health intervention on maternal care utilisation/Battle et al, 2015 | Original NOS | 7/9 | Low risk | It's a mixed methods design, not appropriate for the NOS. However, we evaluated the quantitative component with NOS to maintain consistency |

| | | | | | | |
|---|---|---|---|---|---|---|
| 6 | Cross-sectional | Using Mobile Technology to Address the 'Three Delays' to Reduce Maternal Mortality/Deussom et al, 2016 | Modified NOS | 5/10 | Moderate risk | |
| 7 | Process evaluation | Improving health facility delivery rates in Zanzibar, Tanzania through a large-scale digital community health volunteer programme: a process evaluation/Fulcher et al, 2020 | Modified NOS | 6/10 | Moderate risk | The process evaluation has other tools like. TIDieR or CASP Checklists however, for the quantitative data components, the Modified NOS was used. |
| 8 | Mixed-method, quasi-experimental | Effectiveness of an Electronic Partogram/Sanghvi et al, 2019 | Original NOS | 7/9 | Low risk | Mixed-methods, Quasi experiment, but we adapted the NOS |
| 9.a) | Cross-sectional | The Effects of a Locally Developed mHealth Intervention on Delivery and Postnatal Care Utilization/Shiferaw et al, 2016 | Modified NOS | 6/10 | Moderate risk | It's a mixed methods design employed crosse-sectional and longitudinal follow-up.<br>-This is for cross-section component score |
| b) | Longitudinal follow-up | | Original NOS | 7/9 | Low risk | This scoring for longitudinal component |
| 10 | Pre/post-intervention | Assessment of the quality of antenatal care services provided by health workers using a mobile phone decision support application/McNabb et al, 2015 | Original NOS | 7/9 | Low risk | We adapted NOS for the quantitative component |
| 11 | Cluster randomised control trial | Mobile Clinical Decision Support for the Quality Improvement of Maternal, Neonatal and Child Health Services Delivered by Community Health Workers/Fazen, 2015 | Cochrane Collaboration's Risk of Bias Tool for Cluster Randomized Trials | No numeric scoring | Moderate risk | Lack of blinding and baseline imbalance |

| | | | | | | |
|---|---|---|---|---|---|---|
| 12 | Usability evaluation and pilot | Usability and Feasibility of PIERS on the Move: An mHealth App for Pre-Eclampsia Triage/Lim et al, 2015 | Original NOS | 7/9 | Low risk | Developmental research design focusing on usability/feasibility testing. We adapted NOS for the quantitative component. |
| 13 | Development | Development of mHealth Applications for Pre-Eclampsia Triage/Dunsmuir et al, 2014 | Original NOS | 8/9 | Low risk | It's a developmental research design. The Mixed-methods appraisal tool (MMAT) could do better but for consistency we have to stick to the NOS |
| 14 | Focus groups and in-depth interviews | It makes you someone who changes with the times': health worker and client perspectives on a smartphone-based counselling application/Hackett et al, 2019 | Original NOS | 7/9 | Low risk | The CASP checklist could be appropriate for this qualitative study. But for more consistency, we used NOS since this study was embedded with a Cluster randomized community intervention trial which contained a cohort of quantitative data. |
| 15 | Pilot and descriptive quantitative analysis | Viability of diagnostic decision support for antenatal care in rural settings/Aberjirinde et al, 2019 | Original NOS | 7/9 | Low risk | Embedded study design, we adapted the NOS for the quantitative component |
| 16 | Technology evaluation | Unveiling the Black Box of Diagnostic and Clinical Decision Support Systems for Antenatal Care: Realist Evaluation/Aberjirinde et al, 2018 | Original NOS | 7/9 | Low risk | NOS was not an appropriate method for this design since it's a multiple case combined |

| | | | | | | |
|---|---|---|---|---|---|---|
| | | | | | | with realist evaluation. However, for transparency and to maintain consistency in quality assessment, we considered the key aspects of the NOS i.e. selection, comparability, and outcome assessment. |
| 17 | Mixed - methods design | Pregnant women's experiences with an integrated diagnostic and decision support device for antenatal care/Aberjirinde et al, 2018 | Original NOS | 7/9 | Low risk | It's a mixed methods design. |
| 18 | Pilot study | Usability and feasibility of a mobile health system to provide comprehensive antenatal care in low-income countries/Benski et al, 2017 | Original NOS | 7/9 | Low risk | NOS criteria were adapted to assess the quality of the quantitative component |
| 19 | Questionnaire and interviews | Use of a mHealth System to Improve Antenatal Care in Low and Lower-Middle Income Countries/Paduano et al, 2022 | Original NOS | 8/9 | Low risk | NOS was done for the quantitative component |
| 20 | Cluster randomised control trial protocol | Effects of the Pregnancy and Newborn Diagnostic Assessment (PANDA) App on Antenatal Care Quality in Burkina Faso: Protocol for a Cluster Randomised Controlled Trial/Coulibaly and Kouanda, 2023 | Cochrane Collaboration’s Risk of Bias Tool for Cluster Randomized Trials | No numeric scoring | Moderate risk | Lack of blinding and baseline imbalance |
| 21 | Program evaluation | Community Health Volunteers, Digital Health, and a path towards Safer Deliveries /Said, 2018 | Original NOS | 7/9 | Low risk | The logic model approach was appropriate for this. We adapted NOS for the quantitative component |

| | | | | | | |
|---|---|---|---|---|---|---|
| 22 | Implementation research design | The Journey of Zanzibar's Digitally Enabled Community Health Program to National Scale: Implementation Report | Original NOS | 7/9 | Low risk | RE-AIM Framework is an alternative here, but we adapted the NOS |
| 23 | Mixed methods design | mHealth4Afrika Beta v1 Validation in Rural and Deep Rural Clinics/Cunningham et al, 2018 | Original NOS | 7/9 | Low risk | Mixed-method Appraisal Tool could have worked better here. We adapted NOS |
| 24 | Participatory action research design | mHealth4Afrika - Co-designing an Integrated Solution for Resource Constrained Environments/Cunningham et al, 2018 | Original NOS | 7/9 | Low risk | NOS was adapted, though the RE-AIM Framework could have worked better |
| 25 | Mixed-methods design | mHealth4Afrika Alpha Validation in Rural and Deep Rural Clinics/Cunningham et al, 2017 | Original NOS | 7/9 | Low risk | The NOS was adapted for the quantitative component |
| 26 | Developmental evaluation design | The Development of an Electronic Clinical Decision and Support System to Improve the Quality of Antenatal Care/van Pelt et al, 2021 | Original NOS | 7/9 | Low risk | We adopted the NOS for consistency, though the RE-AIM Framework could be good here |
| 27 | Qualitative study design | "If you don't have enough equipment, you're not going to provide quality services": Healthcare workers' perceptions on improving the quality of antenatal care/van Pelt et al, 2020 | COREQ-Consolidated Criteria for Reporting Qualitative Research | No scoring for COREQ | Based on the COREQ it was a high-quality article to be included in the analysis | This was a purely qualitative study without any quantitative component. Therefore, we could not use the NOS here, instead, we used COREQ |
| 28 | Qualitative research design | Pregnant women's perceptions of antenatal care and utilisation of digital health tools/van Pelt et al, 2023 | COREQ | Not applicable for COREQ | High-quality suitable for analysis | NOS cannot be used here, so we used COREQ |
| 29 | Mixed methods | Use of an electronic Partograph: feasibility and acceptability study/Litwin et al, 2018 | Original NOS | 7/9 | Low risk | We adapted NOS, though The Mixed-methods appraisal tool (MMAT) could be an alternative |

| | | | | | | |
|---|---|---|---|---|---|---|
| 30 | Quasi-experiment design | The role of a decision support smartphone application in enhancing community health volunteers' effectiveness to improve maternal and newborn outcomes in Nairobi, Kenya: quasi experimental research protocol /Bakibinga et al, 2017 | Original NOS | 7/9 | Low risk | We adapted the NOS |
| 31 | Qualitative study design | Challenges and prospects for implementation of community health volunteers' digital health solutions/Bakibinga et al, 2020 | COREQ | Not applicable for COREQ | High-quality suitable for analysis | NOS cannot be used here, so we used COREQ |
| 32 | Mixed methods | Designing mHealth for maternity services in primary health facilities in a low-income setting – lessons from a partially successful implementation/Shiferaw et al, 2018 | Original NOS | 9/9 | Low risk | We adapted the NOS |
| 33 | Descriptive implementation study design | CommCare: Automated Quality Improvement to strengthen community/Svoronos et al, 2014 | Original NOS | 9/9 | Low risk | We adapted the NOS |
| 34 | Qualitative study design | Qualitative assessment of the feasibility, usability and acceptability of a mobile client data app for community based maternal, neonatal and child care/Rothstein et al, 2016 | COREQ | Not applicable for COREQ | High-quality suitable for analysis | NOS cannot be used here, so we used COREQ |
| 35 | Conference abstract | Healthy mama Application: Feasibility, Acceptability and Utility of an Innovative mHealth Intervention to Improve Maternal Child Health Services/Babirye and Passy, 2019 | Not applicable | Not applicable | Not applicable | This one was hard to evaluate since there was no adequate information |
| 36 | Cluster randomised control trial | Mobile Clinical Decision Support for the Quality Improvement of Maternal, Neonatal and Child Health Services Delivered by Community Health Workers/Fazen, 2015 | Cochrane Collaboration's Risk of Bias Tool for Cluster Randomized Trials | No numeric scoring | Moderate risk | Lack of blinding and baseline imbalance |

Key:

NOS= Newcastle-Ottawa Scale; COREQ= Consolidated Criteria for Reporting Qualitative Research; RE-AIM=Reach, Effectiveness, Adoption, Implementation, and Maintenance: TIDieR= Template for Intervention Description and Replication; MMAT= Mixed-Methods Appraisal Tool

# Appendix

## 1. A. Revised Cochrane risk-of-bias tool for randomized trials (RoB 2)
## TEMPLATE FOR COMPLETION

Edited by Julian PT Higgins, Jelena Savović, Matthew J Page, Jonathan AC Sterne
on behalf of the RoB2 Development Group

**Version of 22 August 2019**

The development of the RoB 2 tool was supported by the MRC Network of Hubs for Trials Methodology Research (MR/L004933/2- N61), with the support of the host MRC ConDuCT-II Hub (Collaboration and innovation for Difficult and Complex randomised controlled Trials In Invasive procedures - MR/K025643/1), by MRC research grant MR/M025209/1, and by a grant from The Cochrane Collaboration.

This work is licensed under a Creative Commons Attribution-NonCommercial-NoDerivatives 4.0 International License.

**Study details**

| | |
|---|---|
| **Reference** | |

**Study design**

X Individually-randomized parallel-group trial

☐ Cluster-randomized parallel-group trial
☐ Individually randomized cross-over (or other matched) trial

**For the purposes of this assessment, the interventions being compared are defined as**

Experimental: ______ Comparator: ______

**Specify which outcome is being assessed for risk of bias** ______

**Specify the numerical result being assessed.** In case of multiple alternative analyses being presented, specify the numeric result (e.g. RR = 1.52 (95% CI 0.83 to 2.77) and/or a reference (e.g. to a table, figure or paragraph) that uniquely defines the result being assessed.

**Is the review team's aim for this result…?**

☐ to assess the effect of *assignment to intervention* (the 'intention-to-treat' effect)
☐ to assess the effect of *adhering to intervention* (the 'per-protocol' effect)

**If the aim is to assess the effect of *adhering to intervention***, select the deviations from intended intervention that should be addressed (at least one must be checked):

☐ occurrence of non-protocol interventions
☐ failures in implementing the intervention that could have affected the outcome
☐ non-adherence to their assigned intervention by trial participants

**Which of the following sources were <u>obtained</u> to help inform the risk-of-bias assessment? (tick as many as apply)**

☐ Journal article(s) with results of the trial
☐ Trial protocol
☐ Statistical analysis plan (SAP)
☐ Non-commercial trial registry record (e.g. ClinicalTrials.gov record)
☐ Company-owned trial registry record (e.g. GSK Clinical Study Register record)
☐ "Grey literature" (e.g. unpublished thesis)
☐ Conference abstract(s) about the trial
☐ Regulatory document (e.g. Clinical Study Report, Drug Approval Package)
☐ Research ethics application

| |
|---|
| ☐ Grant database summary (e.g. NIH RePORTER or Research Councils UK Gateway to Research)<br>☐ Personal communication with trialist<br>☐ Personal communication with the sponsor |

## Risk of bias assessment

Responses underlined in green are potential markers for low risk of bias, and responses in red are potential markers for a risk of bias. Where questions relate only to sign posts to other questions, no formatting is used.

### Domain 1: Risk of bias arising from the randomization process

| Signalling questions | Comments | Response options |
|---|---|---|
| **1.1 Was the allocation sequence random?** | | Y / PY / PN / N / NI |
| **1.2 Was the allocation sequence concealed until participants were enrolled and assigned to interventions?** | | Y / PY / PN / N / NI |
| **1.3 Did baseline differences between intervention groups suggest a problem with the randomization process?** | | Y / PY / PN / N / NI |
| **Risk-of-bias judgement** | | Low / High / Some concerns |
| Optional: What is the predicted direction of bias arising from the randomization process? | | NA / Favours experimental / Favours comparator / Towards null /Away from null / Unpredictable |

Domain 2: Risk of bias due to deviations from the intended interventions (*effect of assignment to intervention*)

| Signalling questions | Comments | Response options |
|---|---|---|
| **2.1. Were participants aware of their assigned intervention during the trial?** | | Y / PY / PN / N / NI |
| **2.2. Were carers and people delivering the interventions aware of participants' assigned intervention during the trial?** | | Y / PY / PN / N / NI |
| **2.3. If Y/PY/NI to 2.1 or 2.2: Were there deviations from the intended intervention that arose because of the trial context?** | | NA / Y / PY / PN / N / NI |
| **2.4 If Y/PY to 2.3: Were these deviations likely to have affected the outcome?** | | NA / Y / PY / PN / N / NI |
| **2.5. If Y/PY/NI to 2.4: Were these deviations from intended intervention balanced between groups?** | | NA / Y / PY / PN / N / NI |
| **2.6 Was an appropriate analysis used to estimate the effect of assignment to intervention?** | | Y / PY / PN / N / NI |
| **2.7 If N/PN/NI to 2.6: Was there potential for a substantial impact (on the result) of the failure to analyse participants in the group to which they were randomized?** | | NA / Y / PY / PN / N / NI |
| **Risk-of-bias judgement** | | Low / High / Some concerns |
| Optional: What is the predicted direction of bias due to deviations from intended interventions? | | NA / Favours experimental / Favours comparator / Towards null /Away from null / Unpredictable |

Domain 2: Risk of bias due to deviations from the intended interventions (*effect of adhering to intervention*)

| Signalling questions | Comments | Response options |
|---|---|---|
| **2.1. Were participants aware of their assigned intervention during the trial?** | | Y / PY / PN / N / NI |
| **2.2. Were carers and people delivering the interventions aware of participants' assigned intervention during the trial?** | | Y / PY / PN / N / NI |
| **2.3. [If applicable:] If Y/PY/NI to 2.1 or 2.2: Were important non-protocol interventions balanced across intervention groups?** | | NA / Y / PY / PN / N / NI |
| **2.4. [If applicable:] Were there failures in implementing the intervention that could have affected the outcome?** | | NA / Y / PY / PN / N / NI |
| **2.5. [If applicable:] Was there non-adherence to the assigned intervention regimen that could have affected participants' outcomes?** | | NA / Y / PY / PN / N / NI |
| **2.6. If N/PN/NI to 2.3, or Y/PY/NI to 2.4 or 2.5: Was an appropriate analysis used to estimate the effect of adhering to the intervention?** | | NA / Y / PY / PN / N / NI |
| **Risk-of-bias judgement** | | Low / High / Some concerns |
| Optional: What is the predicted direction of bias due to deviations from intended interventions? | | NA / Favours experimental / Favours comparator / Towards null /Away from null / Unpredictable |

## Domain 3: Missing outcome data

| Signalling questions | Comments | Response options |
|---|---|---|
| **3.1 Were data for this outcome available for all, or nearly all, participants randomized?** | | Y / PY / PN / N / NI |
| **3.2 If N/PN/NI to 3.1: Is there evidence that the result was not biased by missing outcome data?** | | NA / Y / PY / PN / N |
| **3.3 If N/PN to 3.2: Could missingness in the outcome depend on its true value?** | | NA / Y / PY / PN / N / NI |
| **3.4 If Y/PY/NI to 3.3: Is it likely that missingness in the outcome depended on its true value?** | | NA / Y / PY / PN / N / NI |
| **Risk-of-bias judgement** | | Low / High / Some concerns |
| Optional: What is the predicted direction of bias due to missing outcome data? | | NA / Favours experimental / Favours comparator / Towards null /Away from null / Unpredictable |

## Domain 4: Risk of bias in measurement of the outcome

| Signalling questions | Comments | Response options |
|---|---|---|
| **4.1 Was the method of measuring the outcome inappropriate?** | | Y / PY / PN / N / NI |
| **4.2 Could measurement or ascertainment of the outcome have differed between intervention groups?** | | Y / PY / PN / N / NI |
| **4.3 If N/PN/NI to 4.1 and 4.2: Were outcome assessors aware of the intervention received by study participants?** | | NA / Y / PY / PN / N / NI |
| **4.4 If Y/PY/NI to 4.3: Could assessment of the outcome have been influenced by knowledge of intervention received?** | | NA / Y / PY / PN / N / NI |
| **4.5 If Y/PY/NI to 4.4: Is it likely that assessment of the outcome was influenced by knowledge of intervention received?** | | NA / Y / PY / PN / N / NI |
| **Risk-of-bias judgement** | | Low / High / Some concerns |
| Optional: What is the predicted direction of bias in measurement of the outcome? | | NA / Favours experimental / Favours comparator / Towards null /Away from null / Unpredictable |

## Domain 5: Risk of bias in selection of the reported result

| Signalling questions | Comments | Response options |
|---|---|---|
| **5.1 Were the data that produced this result analysed in accordance with a pre-specified analysis plan that was finalized before unblinded outcome data were available for analysis?** | | Y / PY / PN / N / NI |
| **Is the numerical result being assessed likely to have been selected, on the basis of the results, from...** | | |
| **5.2. ... multiple eligible outcome measurements (e.g. scales, definitions, time points) within the outcome domain?** | | Y / PY / PN / N / NI |
| **5.3 ... multiple eligible analyses of the data?** | | Y / PY / PN / N / NI |
| **Risk-of-bias judgement** | | Low / High / Some concerns |
| Optional: What is the predicted direction of bias due to selection of the reported result? | | NA / Favours experimental / Favours comparator / Towards null /Away from null / Unpredictable |

## Overall risk of bias

| | | |
|---|---|---|
| **Risk-of-bias judgement** | | Low / High / Some concerns |
| Optional: What is the overall predicted direction of bias for this outcome? | | NA / Favours experimental / Favours comparator / Towards null /Away from null / Unpredictable |

This work is licensed under a Creative Commons Attribution-NonCommercial-NoDerivatives 4.0 International License

# 1 B. Revised Cochrane risk-of-bias tool for cluster-randomized trials (RoB 2 CRT)
# TEMPLATE FOR COMPLETION

**Version of 18 March 2021**

The development of the RoB 2 tool was supported by the MRC Network of Hubs for Trials Methodology Research (MR/L004933/2- N61), with the support of the host MRC ConDuCT-II Hub (Collaboration and innovation for Difficult and Complex randomised controlled Trials In Invasive procedures - MR/K025643/1), by MRC research grant MR/M025209/1, and by a grant from The Cochrane Collaboration.

This work is licensed under a Creative Commons Attribution-NonCommercial-NoDerivatives 4.0 International License.

**Study details**

**Reference**

**Study design**

☐ Individually-randomized parallel-group trial
X Cluster-randomized parallel-group trial
☐ Individually randomized cross-over (or other matched) trial

**For the purposes of this assessment, the interventions being compared are defined as**

Experimental: ______ Comparator: ______

**Specify which outcome is being assessed for risk of bias** ______

**Specify the numerical result being assessed.** In case of multiple alternative analyses being presented, specify the numeric result (e.g. RR = 1.52 (95% CI 0.83 to 2.77) and/or a reference (e.g. to a table, figure or paragraph) that uniquely defines the result being assessed.

**Is the review team's aim for this result…?**

☐ to assess the effect of *assignment to intervention* (the 'intention-to-treat' effect)
☐ to assess the effect of *adhering to intervention* (the 'per-protocol' effect)

**If the aim is to assess the effect of *adhering to intervention***, select the deviations from intended intervention that should be addressed (at least one must be checked):

☐ occurrence of non-protocol interventions
☐ failures in implementing the intervention that could have affected the outcome
☐ non-adherence to their assigned intervention by trial participants

**Which of the following sources were <u>obtained</u> to help inform the risk-of-bias assessment? (tick as many as apply)**

☐ Journal article(s) with results of the trial
☐ Trial protocol
☐ Statistical analysis plan (SAP)
☐ Non-commercial trial registry record (e.g. ClinicalTrials.gov record)
☐ Company-owned trial registry record (e.g. GSK Clinical Study Register record)
☐ "Grey literature" (e.g. unpublished thesis)
☐ Conference abstract(s) about the trial
☐ Regulatory document (e.g. Clinical Study Report, Drug Approval Package)
☐ Research ethics application
☐ Grant database summary (e.g. NIH RePORTER or Research Councils UK Gateway to Research)
☐ Personal communication with trialist
☐ Personal communication with the sponsor

## Risk of bias assessment

Responses underlined in green are potential markers for low risk of bias, and responses in red are potential markers for a risk of bias. Where questions relate only to sign posts to other questions, no formatting is used.

### Domain 1a: Risk of bias arising from the randomization process

| Signalling questions | Comments | Response options |
|---|---|---|
| **1a.1 Was the allocation sequence random?** | | Y / PY / PN / N / NI |
| **1a.2 Was the allocation sequence concealed until clusters were enrolled and assigned to interventions?** | | Y / PY / PN / N / NI |
| **1a.3 Did baseline differences between intervention groups suggest a problem with the randomization process?** | | Y / PY / PN / N / NI |
| **Risk-of-bias judgement** | | Low / High / Some concerns |
| Optional: What is the predicted direction of bias arising from the randomization process? | | NA / Favours experimental / Favours comparator / Towards null /Away from null / Unpredictable |

## Domain 1b: Risk of bias arising from the timing of identification or recruitment of participants in a cluster-randomized trial

| Signalling questions | Comments | Response options |
|---|---|---|
| **1b.1 Were all the individual participants identified and recruited (if appropriate) before randomization of clusters?** | | Y/PY/PN/N/NI |
| **1b.2 If N/PN/NI to 1b.1: Is it likely that selection of individual participants was affected by knowledge of the intervention assigned to the cluster?** | | NA/Y/PY/PN/N/NI |
| **1b.3 Were there baseline imbalances that suggest differential identification or recruitment of individual participants between intervention groups?** | | Y/PY/PN/N/NI |
| **Risk-of-bias judgement** | | Low / High / Some concerns |
| Optional: What is the predicted direction of bias arising from the timing of identification and recruitment of participants? | | NA / Favours experimental / Favours comparator / Towards null /Away from null / Unpredictable |

Domain 2: Risk of bias due to deviations from the intended interventions (*effect of assignment to intervention*)

| Signalling questions | Comments | Response options |
|---|---|---|
| **2.1a Were participants aware that they were in a trial?** | | Y / PY / PN / N / NI |
| **2.1b. If Y/PY/NI to 2.1a: Were participants aware of their assigned intervention during the trial?** | | NA / Y / PY / PN / N / NI |
| **2.2. Were carers and people delivering the interventions aware of participants' assigned intervention during the trial?** | | Y / PY / PN / N / NI |
| **2.3. If Y/PY/NI to 2.1 or 2.2: Were there deviations from the intended intervention that arose because of the trial context?** | | NA / Y / PY / PN / N / NI |
| **2.4 If Y/PY to 2.3: Were these deviations likely to have affected the outcome?** | | NA / Y / PY / PN / N / NI |
| **2.5. If Y/PY/NI to 2.4: Were these deviations from intended intervention balanced between groups?** | | NA / Y / PY / PN / N / NI |
| **2.6 Was an appropriate analysis used to estimate the effect of assignment to intervention?** | | Y / PY / PN / N / NI |
| **2.7 If N/PN/NI to 2.6: Was there potential for a substantial impact (on the result) of the failure to analyse participants in the group to which they were randomized?** | | NA / Y / PY / PN / N / NI |
| **Risk-of-bias judgement** | | Low / High / Some concerns |
| Optional: What is the predicted direction of bias due to deviations from intended interventions? | | NA / Favours experimental / Favours comparator / Towards null /Away from null / Unpredictable |

Domain 2: Risk of bias due to deviations from the intended interventions (*effect of adhering to intervention*)

| **Signalling questions** | **Comments** | **Response options** |
|---|---|---|
| **2.1. Were participants aware of their assigned intervention during the trial?** | | Y / PY / PN / N / NI |
| **2.2. Were carers and people delivering the interventions aware of participants' assigned intervention during the trial?** | | Y / PY / PN / N / NI |
| **2.3. [If applicable:] If Y/PY/NI to 2.1 or 2.2: Were important non-protocol interventions balanced across intervention groups?** | | NA / Y / PY / PN / N / NI |
| **2.4. [If applicable:] Were there failures in implementing the intervention that could have affected the outcome?** | | NA / Y / PY / PN / N / NI |
| **2.5. [If applicable:] Was there non-adherence to the assigned intervention regimen that could have affected participants' outcomes?** | | NA / Y / PY / PN / N / NI |
| **2.6. If N/PN/NI to 2.3, or Y/PY/NI to 2.4 or 2.5: Was an appropriate analysis used to estimate the effect of adhering to the intervention?** | | NA / Y / PY / PN / N / NI |
| **Risk-of-bias judgement** | | Low / High / Some concerns |
| Optional: What is the predicted direction of bias due to deviations from intended interventions? | | NA / Favours experimental / Favours comparator / Towards null /Away from null / Unpredictable |

Domain 3: Risk of bias due to missing outcome data

| Signalling questions | Comments | Response options |
|---|---|---|
| **3.1a Were data for this outcome available for all clusters that recruited participants?** | | Y / PY / PN / N / NI |
| **3.1b Were data for this outcome available for all, or nearly all, participants within clusters?** | | Y / PY / PN / N / NI |
| **3.2 If N/PN/NI to 3.1a or 3.1b: Is there evidence that the result was not biased by missing data?** | | NA / Y / PY / PN / N |
| **3.3 If N/PN to 3.2 Could missingness in the outcome depend on its true value?** | | NA / Y / PY / PN / N / NI |
| **3.4 If Y/PY/NI to 3.3: Is it likely that missingness in the outcome depended on its true value?** | | NA / Y / PY / PN / N / NI |
| **Risk-of-bias judgement** | | Low / High / Some concerns |
| Optional: What is the predicted direction of bias due to missing outcome data? | | NA / Favours experimental / Favours comparator / Towards null /Away from null / Unpredictable |

Domain 4: Risk of bias in measurement of the outcome

| Signalling questions | Comments | Response options |
|---|---|---|
| **4.1 Was the method of measuring the outcome inappropriate?** | | Y / PY / PN / N / NI |
| **4.2 Could measurement or ascertainment of the outcome have differed between intervention groups?** | | Y / PY / PN / N / NI |
| **4.3a If N/PN/NI to 4.1 and 4.2: Were outcome assessors aware that a trial was taking place?** | | NA / Y / PY / PN / N / NI |
| **4.3b If Y/PY/NI to 4.3a: Were outcome assessors aware of the intervention received by study participants?** | | NA / Y / PY / PN / N / NI |
| **4.4 If Y/PY/NI to 4.3b: Could assessment of the outcome have been influenced by knowledge of intervention received?** | | NA / Y / PY / PN / N / NI |
| **4.5 If Y/PY/NI to 4.4: Is it likely that assessment of the outcome was influenced by knowledge of intervention received?** | | NA / Y / PY / PN / N / NI |
| **Risk-of-bias judgement** | | Low / High / Some concerns |
| Optional: What is the predicted direction of bias in measurement of the outcome? | | NA / Favours experimental / Favours comparator / Towards null /Away from null / Unpredictable |

## Domain 5: Risk of bias in selection of the reported result

| Signalling questions | Comments | Response options |
|---|---|---|
| **5.1 Were the data that produced this result analysed in accordance with a pre-specified analysis plan that was finalized before unblinded outcome data were available for analysis?** | | Y / PY / PN / N / NI |
| **Is the numerical result being assessed likely to have been selected, on the basis of the results, from...** | | |
| **5.2. ... multiple eligible outcome measurements (e.g. scales, definitions, time points) within the outcome domain?** | | Y / PY / PN / N / NI |
| **5.3 ... multiple eligible analyses of the data?** | | Y / PY / PN / N / NI |
| **Risk-of-bias judgement** | | Low / High / Some concerns |
| Optional: What is the predicted direction of bias due to selection of the reported result? | | NA / Favours experimental / Favours comparator / Towards null /Away from null / Unpredictable |

## Overall risk of bias

| | | |
|---|---|---|
| **Risk-of-bias judgement** | | Low / High / Some concerns |
| Optional: What is the overall predicted direction of bias for this outcome? | | NA / Favours experimental / Favours comparator / Towards null /Away from null / Unpredictable |

This work is licensed under a Creative Commons Attribution-NonCommercial-NoDerivatives 4.0 International License

Appendix 2.0

**Consolidated criteria for reporting qualitative studies (COREQ): 32-item checklist**

| **No. Item** | **Guide questions/description** | **Reported on Page #** |
|---|---|---|
| **Domain 1: Research team and reflexivity** | | |
| *Personal Characteristics* | | |
| 1. Interviewer/facilitator | Which author/s conducted the interview or focus group? | |
| 2. Credentials | What were the researcher's credentials? E.g. PhD, MD | |
| 3. Occupation | What was their occupation at the time of the study? | |
| 4. Gender | Was the researcher male or female? | |
| 5. Experience and training | What experience or training did the researcher have? | |
| *Relationship with participants* | | |
| 6. Relationship established | Was a relationship established prior to study commencement? | |
| 7. Participant knowledge of the interviewer | What did the participants know about the researcher? e.g. personal goals, reasons for doing the research | |
| 8. Interviewer characteristics | What characteristics were reported about the inter viewer/facilitator? e.g. Bias, assumptions, reasons and interests in the research topic | |

| | | |
|---|---|---|
| **Domain 2: study design** | | |
| *Theoretical framework* | | |
| 9. Methodological orientation and Theory | What methodological orientation was stated to underpin the study? e.g. grounded theory, discourse analysis, ethnography, phenomenology, content analysis | |
| *Participant selection* | | |
| 10. Sampling | How were participants selected? e.g. purposive, convenience, consecutive, snowball | |
| 11. Method of approach | How were participants approached? e.g. face-to-face, telephone, mail, email | |
| 12. Sample size | How many participants were in the study? | |
| 13. Non-participation | How many people refused to participate or dropped out? Reasons? | |
| *Setting* | | |
| 14. Setting of data collection | Where was the data collected? e.g. home, clinic, workplace | |
| 15. Presence of non-participants | Was anyone else present besides the participants and researchers? | |
| 16. Description of sample | What are the important characteristics of the sample? e.g. demographic data, date | |
| *Data collection* | | |
| 17. Interview guide | Were questions, prompts, guides provided by the authors? Was it pilot tested? | |
| 18. Repeat interviews | Were repeat inter views carried out? If yes, how many? | |
| 19. Audio/visual recording | Did the research use audio or visual recording to collect the data? | |
| 20. Field notes | Were field notes made during and/or after the interview or focus group? | |
| 21. Duration | What was the duration of the inter views or focus group? | |
| 22. Data saturation | Was data saturation discussed? | |
| 23. Transcripts returned | Were transcripts returned to participants for comment and/or correction? | |
| **Domain 3: analysis and findings** | | |
| *Data analysis* | | |
| 24. Number of data coders | How many data coders coded the data? | |

| | | |
|---|---|---|
| 25. Description of the coding tree | Did authors provide a description of the coding tree? | |
| 26. Derivation of themes | Were themes identified in advance or derived from the data? | |
| 27. Software | What software, if applicable, was used to manage the data? | |
| 28. Participant checking | Did participants provide feedback on the findings? | |
| *Reporting* | | |
| 29. Quotations presented | Were participant quotations presented to illustrate the themes/findings? Was each quotation identified? e.g. participant number | |
| 30. Data and findings consistent | Was there consistency between the data presented and the findings? | |
| 31. Clarity of major themes | Were major themes clearly presented in the findings? | |
| 32. Clarity of minor themes | Is there a description of diverse cases or discussion of minor themes? | |

## Appendix 3.0

**Newcastle-Ottawa Scale adapted for cross-sectional studies**

### Selection:

1. Representativeness of the sample:
   a. Truly representative of the average in the target population. * (all subjects or random sampling)
   b. Somewhat representative of the average in the target group. * (non-random sampling)
   c. Selected group of users/convenience sample.
   d. No description of the derivation of the included subjects.

2. Sample size:
   a. Justified and satisfactory (including sample size calculation). *
   b. Not justified.
   c. No information provided

3. Non-respondents:
   a. Proportion of target sample recruited attains pre-specified target or basic summary of non-respondent characteristics in sampling frame recorded. *
   b. Unsatisfactory recruitment rate, no summary data on non-respondents.
   c. No information provided

4. Ascertainment of the exposure (risk factor):

   a. Vaccine records/vaccine registry/clinic registers/hospital records only. **
   b. Parental or personal recall and vaccine/hospital records. *
   c. Parental/personal recall only.

**Comparability:** (Maximum 2 stars)

1. Comparability of subjects in different outcome groups on the basis of design or analysis. Confounding factors controlled.
   a. Data/ results adjusted for relevant predictors/risk factors/confounders e.g. age, sex, time since vaccination, etc. **
   b. Data/results not adjusted for all relevant confounders/risk factors/information not provided.

Outcome:

1. Assessment of outcome:
   a. Independent blind assessment using objective validated laboratory methods. **
   b. Unblinded assessment using objective validated laboratory methods. **
   c. Used non-standard or non-validated laboratory methods with gold standard. *
   d. No description/non-standard laboratory methods used.

2. Statistical test:
   a. Statistical test used to analyse the data clearly described, appropriate and measures of association presented including confidence intervals and probability level (p value). *
   b. Statistical test not appropriate, not described or incomplete.

Cross-sectional Studies:
Very Good Studies: 9-10 points
Good Studies: 7-8 points
Satisfactory Studies: 5-6 points
Unsatisfactory Studies: 0 to 4 points

This scale has been adapted from the Newcastle-Ottawa Quality Assessment Scale for cohort studies to provide a quality assessment of cross-sectional studies[1].

[1] Herzog R, et al. Is Healthcare Workers' Intention to Vaccinate Related to their Knowledge, Beliefs and Attitudes? A Systematic Review. *BMC Public Health* 2013 **13**:154
