# Supplementary material for: The mHealth clinical decision-making tools for maternal and perinatal health care in Sub-Saharan Africa: A systematic review
Source: PLoS One. 2025 Apr 24;20(4):e0319510. doi: 10.1371/journal.pone.0319510 (PMC12021198; doi:10.1371/journal.pone.0319510)
Supplement: S4 File — (PDF) [file pone.0319510.s004.pdf]

Table 1A Articles describing the quantitative impact of mHealth clinical decision-making tools on pregnancy-related outcomes

Article title/Author, Year	Technology	Country	Design	Number of healthcare workers	Number of women	Feasibility	Usability	Acceptability	User satisfaction	Impact on pregnancy outcomes*
The ability and safety of community-based health workers to safely initiate lifesaving therapies for pre-eclampsia in Ogun State, Nigeria: An analysis of 260 community treatments with MgSO4 and/or methyldopa/Adepoju et al, 2021	POTM	Nigeria	Cluster randomised controlled trial	170 CHWs	8790	🟢	🔴	🔴	🔴	🔴
Community-level interventions for pre-eclampsia (CLIP) in Mozambique: A cluster randomised controlled trial/Sevene et al, 2020	POTM	Mozambique	Cluster randomised controlled trial	50 CHWs in intervention arm, NNS control arm	15,013	🔴	🔴	🔴	🔴	🟢
Impact of smartphone-assisted prenatal home visits on women's use of facility delivery: Results from a cluster-randomised trial in rural Tanzania/Hackett et al, 2018	SUSTAIN	Tanzania	Cluster randomised controlled trial	64 CHWs	572	🔴	🔴	🔴	🔴	🟢
Improving the Quality of Antenatal Care Using Mobile Health in Madagascar: Five-Year Cross-Sectional Study/Benski et al, 2020	PANDA	Madagascar	Pre/Post intervention	13 Healthcare providers	1446	🔴	🔴	🔴	🔴	🟢
mHealth for Safer Deliveries: A mixed methods evaluation of the effect of an integrated mobile health intervention on maternal care utilisation/Battle et al, 2015	mHealth for Safer Deliveries	Tanzania	Mixed methods evaluation	223 CHWs	13,231	🔴	🟢	🟢	🟢	🟢
Using Mobile Technology to Address the 'Three Delays' to Reduce Maternal Mortality/Deussom et al, 2016	mHealth for Safer Deliveries	Tanzania	Cross-sectional	24 TBAs	938	🔴	🔴	🟢	🟢	🟢

Improving health facility delivery rates in Zanzibar, Tanzania through a large-scale digital community health volunteer programme: a process evaluation/Fulcher et al, 2020	mHealth for Safer Deliveries	Tanzania	Process evaluation	436 CHWs	41,653	● (green)	● (red)	● (red)	● (red)	● (green)
Effectiveness of an Electronic Partogram/Sanghvi et al, 2019	ePartogram	Kenya	Mixed-method, quasi-experimental	SBAs, NNS	842 interventions, 1042 control	● (green)	● (red)	● (green)	● (green)	● (green)
The Effects of a Locally Developed mHealth Intervention on Delivery and Postnatal Care Utilization/Shiferaw et al, 2016	mHealth system‡	Ethiopia	Cross-sectional	15 HCWs	933 at baseline, 1037 at follow-up	● (red)	● (red)	● (red)	● (red)	● (green)
Assessment of the quality of antenatal care services provided by health workers using a mobile phone decision support application/McNabb et al, 2015	m4Change	Nigeria	Pre/post-intervention	CHEWs and HCWs, NNS	266	● (red)	● (red)	● (green)	● (green)	● (green)
Mobile Clinical Decision Support for the Quality Improvement of Maternal, Neonatal and Child Health Services Delivered by Community Health Workers/Fazen, 2015	Clinical Decision Support (CDS)	Kenya	Cluster randomised control trial	78 CHVs	2974 women and children	● (red)	● (red)	● (green)	● (red)	● (green)

* This includes facility-based delivery rates, number of antenatal/postnatal care visits, maternal and neonatal morbidity and mortality and other reported quantitative pregnancy outcomes.

‡The mHealth app was not given a specific name by the authors (author correspondence).

Key: Described=● (green), Partially Described = ● (yellow), Not Described = ● (red)

POTM = PIERS (Pre-eclampsia Integrated Estimate of RiSk) On the Move; SUSTAIN = Supporting Systems to Improve Nutrition, Maternal, Newborn and Child Health; B4M = Bliss4Midwives; PANDA = Pregnancy and Newborn Diagnostic Assessment; CHW = community health worker; TBA = traditional birth attendant; SBA = skilled birth attendant; CHEW = community health extension worker; HCW = health care worker; CHV = community health volunteer; NNS = number not stated

Table 1B Articles describing the qualitative impacts of mHealth clinical decision-making tools

Article title/Author, Year	Technology	Country	Design	Number of Healthcare Workers	Numbe r of women	Feasi bility	Usabi lity	Acce ptabil ity	User satisf action	*Technology impact on pregnancy outcomes
Usability and Feasibility of PIERS on the Move: An mHealth App for Pre-Eclampsia Triage/Lim et al, 2015	POTM	South Africa	Usability evaluation and pilot	37 nurses and midwives	>200	🔴	🟢	🔴	🟢	🔴
Development of mHealth Applications for Pre-Eclampsia Triage/Dunsmuir et al, 2014	POTM	N/A	Development	c-HCPs, NNS	N/A	🟢	🟢	🔴	🔴	🔴
It makes you someone who changes with the times': health worker and client perspectives on a smartphone-based counselling application/Hackett et al, 2019	SUSTAIN	Tanzania	Focus groups and in-depth interviews	14 CHW supervisors	56	🔴	🔴	🟢	🔴	🔴
Viability of diagnostic decision support for antenatal care in rural settings/Aberjirinde et al, 2019	B4M	Ghana	Pilot and descriptive quantitative analysis	25 midwives and CHWs	940	🟢	🟢	🔴	🔴	🔴
Unveiling the Black Box of Diagnostic and Clinical Decision Support Systems for Antenatal Care: Realist Evaluation/Aberjirinde et al, 2018	B4M	Ghana	Technology evaluation	25 maternal health workers	N/A	🟢	🟢	🟢	🟢	🔴
Pregnant women's experiences with an integrated diagnostic and decision support device for antenatal care/Aberjirinde et al, 2018	B4M	Ghana	Semi-structured interviews and observation	10 HCWs	20	🔴	🔴	🟢	🟢	🟡
Usability and feasibility of a mobile health system to provide comprehensive antenatal care in low-income countries/Benski et al, 2017	PANDA	Madagascar	Pilot study	HCWs, NNS	100	🟢	🟢	🟢	🔴	🔴

Use of a mHealth System to Improve Antenatal Care in Low and Lower-Middle Income Countries/Paduano et al, 2022	PANDA	Tanzania	Questionnaire and interviews	5 HCWs	98	🟡	🟡	🟢	🟢	🔴
Effects of the Pregnancy and Newborn Diagnostic Assessment (PANDA) App on Antenatal Care Quality in Burkina Faso: Protocol for a Cluster Randomised Controlled Trial/Coulibaly and Kouanda, 2023	PANDA	Burkina Faso	Cluster randomised control trial protocol	NA	NA	🔴	🔴	🟡	🟡	🟡
mHealth for Safer Deliveries: A mixed methods evaluation of the effect of an integrated mobile health intervention on maternal care utilization/Battle et al, 2015	mHealth for Safer Deliveries	Tanzania	Mixed methods evaluation	223 CHWs	13,231	🔴	🟢	🟢	🟢	🟢
Community Health Volunteers, Digital Health, and a path towards Safer Deliveries /Said, 2018	mHealth for Safer Deliveries	Tanzania	Web article	N/A	N/A	🔴	🔴	🟢	🟢	🔴
The Journey of Zanzibar's Digitally Enabled Community Health Program to National Scale: Implementation Report	Jamii ni Afya (previously Safer Deliveries)	Tanzania	Implementation Report	2,300 CHWs	>320,000 women	🟢	🟢	🔴	🟡	🟡
mHealth4Afrika Beta v1 Validation in Rural and Deep Rural Clinics/Cunningham et al, 2018	mHealth4Afrika	Ethiopia, Kenya, Malawi & South Africa	Validation study	36 HCWs	NNS	🟢	🟢	🟢	🟡	🔴
mHealth4Afrika - Co-designing an Integrated Solution for Resource Constrained Environments/Cunningham et al, 2018	mHealth4Afrika	Ethiopia, Kenya, Malawi & South Africa	Design and development	N/A	N/A	🟡	🔴	🔴	🔴	🔴
mHealth4Afrika Alpha Validation in Rural and Deep Rural Clinics/Cunningham et al, 2017	mHealth4Afrika	Ethiopia, Kenya, Malawi & South Africa	Validation study	49 HCWs	N/A	🟡	🟢	🔴	🟡	🔴

The Development of an Electronic Clinical Decision and Support System to Improve the Quality of Antenatal Care/van Pelt et al, 2021	Nurse Assistant App	Tanzania	Intervention mapping and implementation	HCWs, NNS	N/A	● (green)	● (green)	● (red)	● (red)	● (red)
"If you don't have enough equipment, you're not going to provide quality services": Healthcare workers' perceptions on improving the quality of antenatal care/van Pelt et al, 2020	Nurse Assistant App	Tanzania	Semi-structured interviews	16 HCWs	N/A	● (red)	● (yellow)	● (green)	● (red)	● (red)
Pregnant women's perceptions of antenatal care and utilisation of digital health tools/van Pelt et al, 2023	Not stated	Tanzania	Semi-structured interviews	N/A	19	● (red)	● (red)	● (green)	● (red)	● (red)
Use of an electronic Partograph: feasibility and acceptability study/Litwin et al, 2018	ePartogram	Tanzania	Observation and short interviews	23 SBAs for observation	103	● (green)	● (green)	● (green)	● (green)	● (red)
The role of a decision support smartphone application in enhancing community health volunteers' effectiveness to improve maternal and newborn outcomes in Nairobi, Kenya: quasi experimental research protocol /Bakibinga et al, 2017	mPAMANECH	Kenya	Research protocol†	CHVs, NNS	N/A	● (red)	● (red)	● (red)	● (red)	● (red)
Challenges and prospects for implementation of community health volunteers' digital health solutions/Bakibinga et al, 2020	mPAMANECH	Kenya	Interviews and focus group discussion	10 CHVs, 15 health providers and sCHMTs	N/A	● (green)	● (green)	● (green)	● (red)	● (red)
Designing mHealth for maternity services in primary health facilities in a low-income setting – lessons from a partially successful implementation/Shiferaw et al, 2018	mHealth system‡	Ethiopia	Pilot testing	15 HCWs	NNS	● (green)	● (green)	● (red)	● (yellow)	● (red)
CommCare: Automated Quality Improvement to strengthen community/Svoronos et al, 2014	CommCare	Tanzania	Pilot	5 CHWs	60	● (yellow)	● (yellow)	● (yellow)	● (yellow)	● (red)

Qualitative assessment of the feasibility, usability and acceptability of a mobile client data app for community based maternal, neonatal and child care/Rothstein et al, 2016	Client Data App	Ghana	Interviews and focus group discussion	8 CHNs, 2 midwives, 4 district health officers interviewed; 15 CHNs in focus groups	N/A	🟢	🟢	🟢	🟢	🔴
Healthy mama Application: Feasibility, Acceptability and Utility of an Innovative mHealth Intervention to Improve Maternal Child Health Services/Babirye and Passy, 2019	Healthy mama	Uganda	Conference abstract†	Healthcare workers, NNS	N/A	🟡	🟡	🟡	🟡	🔴
Mobile Clinical Decision Support for the Quality Improvement of Maternal, Neonatal and Child Health Services Delivered by Community Health Workers/Fazen, 2015	Clinical Decision Support (CDS)	Kenya	Cluster randomised control trial	78 CHVs	2974 woman and children	🔴	🔴	🟢	🔴	🟢

* This includes facility-based delivery rates, number of antenatal/postnatal care visits, maternal and neonatal morbidity and mortality and other reported quantitative pregnancy outcomes

†Authors were contacted, full study data was not published.

Key: Described=🟢, Partially Described = 🟡, Not Described = 🔴

POTM = PIERS (Pre-eclampsia Integrated Estimate of RiSk) On the Move; SUSTAIN = Supporting Systems to Improve Nutrition, Maternal, Newborn and Child Health; B4M = Bliss4Midwives; PANDA = Pregnancy and Newborn Diagnostic Assessment;; N/A = Not Applicable; c-HCP = community-based health care provider; CHW = community health worker; TBA = traditional birth attendant; SBA = skilled birth attendant; CHEW = community health extension worker; HCW = health care worker; CHV = community health volunteer; sCHMT = sub-County Health Management team member; CHN = community health nurse; NNS = number not stated
