# Supplementary material for: The mHealth clinical decision-making tools for maternal and perinatal health care in Sub-Saharan Africa: A systematic review
Source: PLoS One. 2025 Apr 24;20(4):e0319510. doi: 10.1371/journal.pone.0319510 (PMC12021198; doi:10.1371/journal.pone.0319510)
Supplement: S5 File — (PDF) [file pone.0319510.s005.pdf]

Table 2: Technical aspect and intervention content

Freq.	Name	Technology developer	Infrastructure	Data Storage	Interoperability/ HIS context	Intervention content
5	mHealth for Safer Deliveries	D-Tree	Java enabled Nokia phone	Local and online data storage	Use of DHIS2, to interact with existing HMIS	Data collection, decision support, counselling on danger signs, screening for complications, referral to health facility, mobile banking for patient transport
4	POTM	University of British Columbia, Canada; JM Ansermino; P von Dadelszen	Either iPod Touch, iPhone 3 or Android phone, plus pulse oximeter	Local and online data storage	NS	Pre-eclampsia risk prediction - stratified women into usual ante/postnatal care, non-urgent referral or urgent referral, with recommendations made on use of medication
4	PANDA	Servizi Computerizzati, Terre Innovative Healthcare	Android tablet, blood pressure device, tests for diabetes, HIV, syphilis, malaria, anaemia	Local and online data storage	NS	Data collection for antenatal visits, patient education, alerts for abnormal results, web-database with patient records for referral hospitals to use
3*	SUSTAIN	World Vision and D-Tree	Nokia smartphones	Local data storage	NS	Client registration and data management; scheduling visits; prompts for referrals and follow up; decision tree protocols to guide household visits; identification of danger signs
3	B4M	Relitech, Enviu, Simavi, Cordaid, TNO Organization for Applied Scientific Research	Android tablet, pulse oximeter, blood pressure device, urine analyser	Local data storage	NS	Decision support algorithm based on diagnostic tests for pre-eclampsia, gestational diabetes and anaemia
3	mHealth4Afrika	NS	NS	NS	Use of DHIS2, to interact with existing HMIS	Data collection to create electronic health records, input test results, appointment organisation
3	Nurse Assistant App	ICT Healthcare Technology Solutions	Tablet	Local data storage	NS	Data collection during antenatal visits, alerts for abnormalities, visit summary generation, decision support for treatment, referral, follow-up
2	ePartogram	Jhpiego	Android tablet	Local data storage	NS	Labour monitoring, alerts for abnormal results

2	mPAMANECH	NS	Mobile phone	Local and online data storage	NS	Data collection, alerts for danger signs, referral to facilities, data viewable at facilities via web access
2	mHealth system‡	Authors	Android phone	Local and online data storage	NS	Data collection, appointment reminders, decision support system for referrals, education, interaction with web system for data aggregation and report generation
1	CommCare	NS	Nokia phone	Local and online data storage	NS	Data collection for registration, follow up and referral of women; education and counselling; decision support algorithm
1	m4Change	Pathfinder International	Nokia phone	NS	NS	Data collection, decision support algorithm to screen for PET and obstetric danger signs, recommendations for follow up and referral, health counselling via audio messages
1	Client Data App	MOTECH	Nokia phone	Local and online data storage	NS	Data collection, appointment reminders, report generation
1	Healthy mama	NS	NS	NS	NS	Data collection
1	Clinical Decision Support	Author	Android phone	NS	Connection with OpenMRS medical records	Data collection, decision support algorithm to guide follow-up, education resources

*3 papers are published articles, included in the table above. 1 is a PhD thesis from which the manuscripts were published, but also included additional information on the technology and its development.

‡The mHealth app was not given a specific name by the authors (author correspondence).

Key: POTM = PIERS (Pre-eclampsia Integrated Estimate of RiSk) On the Move; SUSTAIN = Supporting Systems to Improve Nutrition, Maternal, Newborn and Child Health; B4M = Bliss4Midwives; PANDA = Pregnancy and Newborn Diagnostic Assessment; NAA = Nurse Assistant App; NS = Not State
